# Multicenter Study of Comorbidities in Patients with Periprosthetic Fractures After Total Hip Arthroplasty and Their Association with Immediate Postoperative Complications

**DOI:** 10.3390/clinpract15060110

**Published:** 2025-06-12

**Authors:** Victor Niculescu, Alexandru Lisias Dimitriu, Delia Carmen Nistor-Cseppento, Sebastian Tirla, Anamaria Gherle, Bogdan Uivaraseanu, Cristian Burnei

**Affiliations:** 1 University Doctoral School, “Carol Davila” University of Medicine and Pharmacy, 050074 Bucharest, Romania; victor.niculescu@drd.umfcd.ro; 2 Department 14 Orthopedics-Intensive Care, “Carol Davila” University of Medicine and Pharmacy, 050474 Bucharest, Romania; cristian.burnei@umfcd.ro; 3 Doctoral School of Biomedical Sciences, Faculty of Medicine and Pharmacy, University of Oradea, 410087 Oradea, Romania; tirla.sebastian@student.uoradea.ro (S.T.);; 4 Department of Psycho-Neuroscience and Recovery, Faculty of Medicine and Pharmacy, University of Oradea, 410073 Oradea, Romania; 5 Department of Surgery, Faculty of Medicine and Pharmacy, University of Oradea, 410073 Oradea, Romania; buivaraseanu@uoradea.ro

**Keywords:** periprosthetic fractures, total hip arthroplasty, comorbidities, post-operative complications

## Abstract

**Background/Objectives:** Periprosthetic fractures (PFs) can occur in both the upper and lower limbs, commonly resulting from falls at the same level. The frequency of PFs following total hip arthroplasty (THA) ranges from 0.045% to 4.1%, and this incidence is influenced by several factors, including age, gender, the type of prosthesis used, and existing comorbidities. Previous studies on this subject have been small in scale and did not adequately address the associated comorbidities, which pose a challenge for the aging population. This study aims to comparatively assess the incidence of THA-related PFs, immediate postoperative complications, and comorbidities in patients with PFs from three emergency hospitals. **Methods:** A retrospective observational study was conducted from 1 January to 31 December 2024, in which 54 patients with PFs hospitalized in three emergency hospitals (Bucharest, Oradea, and Ploiești) were evaluated, divided into Group B (*n* = 29), Group O (*n* = 14), and Group P (*n* = 11). **Results:** Of all patients with PFs, 81.48% had minor complications—grade 1, 9.26% had grade 2 complications (complications requiring medical treatment or other minor interventions), and 3.70% had complications requiring surgery or invasive procedures. Clavien–Dindo grade 5 (patient death) had an incidence of 3.70%. Cardiac pathology was the most common pathology; hypertension predominated in Group O (42.85%). Alzheimer’s disease was associated in 7 patients (12.96%). Without associated pathology, about 13% of patients were identified. Diabetes mellitus also occurred frequently in 31.50%. Data analysis indicates a very weak positive correlation between the Dindo Index and the Charlson Comorbidity Index (r = 0.046), which is not statistically significant (*p* = 0.628). The effect size, measured by Fisher’s z, is also reported as 0.046. **Conclusions:** No significant differences were found among the evaluated centers regarding therapeutic approaches, postoperative complications, and associated comorbidities. Furthermore, there is insufficient evidence to suggest a significant association between the Charlson Comorbidity Index and the Clavien–Dindo Index.

## 1. Introduction

Periprosthetic fractures (PFs) can occur at multiple locations in both the upper and lower extremities, but they are significantly more common in the hip region (over 50%) [1]. These fractures pose a serious challenge in orthopedic practice, highlighting the need for careful management and prevention strategies [2]. The most common cause of these fractures is a fall from the same level, accounting for 76% of cases. In the upper limb, 73% of fractures occur during surgery. The risk of fractures also increases during the removal of implants, making the timing of the intervention critical and necessitating special attention. Additionally, spontaneous fractures are frequently seen after arthroplasty revision due to reduced bone density [3].

PFs associated with total hip arthroplasty (THA) were first reported by Horwitz and Lenobel in 1954. The frequency of these fractures varies significantly, ranging from 0.045% to 4.1%. Several risk factors influence this variability, including gender, age (particularly for individuals over 70 years), the type of prosthesis used (cemented or cementless), the stability of the implant, femoral stem loosening, osteoporosis, and the use of bisphosphonates [3]. Research on age and gender as risk factors for peri-prosthetic femoral fractures after total hip arthroplasty (THA) is inconsistent. Some studies indicate that the risk of periprosthetic fractures is higher, similar, or lower in women compared to men [4].

One registry study suggests that those of age over 70 and female sex are at risk for these fractures, but most of the research is retrospective and small in size, unadjusted for confounding factors. Also, evidence about other risk factors, such as implant types or fracture history, is limited, and no previous studies have analyzed comorbidity (cardio-respiratory, neurological, musculoskeletal disorders, and diabetes mellitus) as a risk factor. Increasing comorbidity in the aging population is a health challenge. In the context of expanding indications for THA, it is essential to understand the impact of comorbidity and body mass index on periprosthetic fractures, which emphasizes the need for robust studies in this area [5].

**Figure 1:**
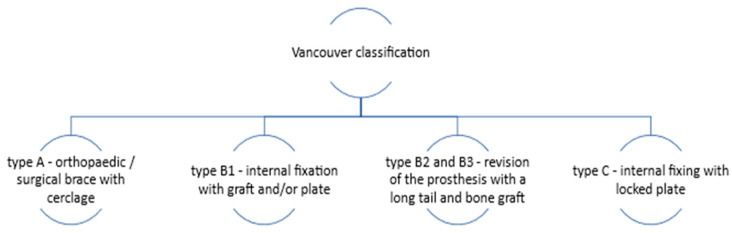
The treatment of PFs according to the Vancouver classification.

The standard treatment for THA-associated PFs involves surgical procedures such as internal fixation or revision arthroplasty (a therapeutic procedure used to replace a previous arthroplasty), which depend on the Vancouver classification of the fracture (Figure 1). This classification is based on the location of the fracture, the stability of the prosthesis, and the quality of the surrounding bone. If the femoral component is stable, open reduction and internal fixation are recommended. An overview of the therapeutic approach according to the Vancouver classification is illustrated in Figure 1 [6,7,8].

The immediate complications associated with surgery include wound issues, which may be accompanied by infections (either superficial or deep), deep vein thrombosis, pulmonary embolism, sciatic nerve palsy, prosthesis dislocation, and refracture, as after primary THA intervention [9]. The incidence of these complications tends to increase with age. The mortality associated with arthroplasty procedures is less than 2.5% during hospitalization. The literature suggests a significant increase in distant mortality (3.5% at 30 days, 4.8% at 90 days, and 13.4% at one year) [10].

The study aims to analyze the incidence of THA-associated PFs, immediate postoperative complications, and comorbidities in patients with PFs, THA-associated, from three emergency hospitals in different areas of Romania. A secondary aim of the study is to identify the relationship between preoperative comorbidities, as assessed by the Deyo–Charlson Index, which is considered a valid measure for assessing comorbidities, and immediate postoperative complications [11].

## 2. Materials and Methods

### 2.1. Database

A retrospective observational study was conducted from 1 January to 31 December 2024, evaluating 54 patients with PFs who were hospitalized in three hospitals in Romania: the Bihor County Emergency Hospital, the Ploiești County Emergency Hospital, and the Bucharest Clinical Emergency Hospital. The patients were categorized based on the hospital to which they were referred, resulting in three groups:

- Group B—patients belonging to Bucharest Emergency Hospital;
- Group O—patients who were referred to the Bihor Clinical Emergency Hospital;
- Group P—patients who were treated in the Ploiești County Emergency Hospital.

#### Inclusion/Exclusion Criteria

All cases of THA-associated PFs within the time frame considered for the study (January–December 2024) were evaluated; there were no exclusion criteria within this group.

**Figure 2:**
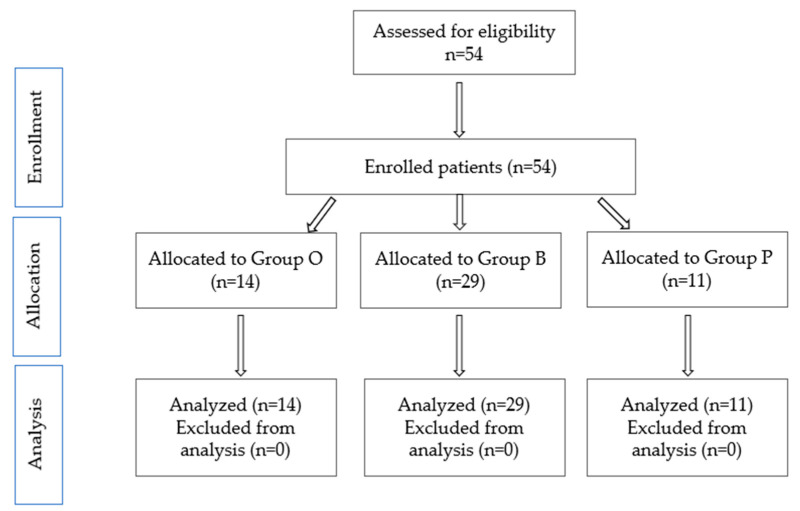
The CONSORT flow diagram of the study.

Figure 2 describes the distribution by study groups.

### 2.2. Study Tools

Risk factors, comorbidities, postoperative complications during hospitalization, type of fracture, therapeutic management, and postoperative outcome during hospitalization were evaluated.

The Deyo–Charlson Comorbidity Index (CCI) was used to quantify comorbidities. Comorbidities were grouped according to previous clinical decisions to simplify the analysis, including heart disease (myocardial infarction, heart failure), peripheral vascular disease, cerebrovascular disease, hemiplegia/paraplegia, moderate to severe renal disease, peptic ulcer, chronic obstructive pulmonary disease, diabetes (with or without impairment), and various types of cancer, as well as other conditions (dementia, liver disease, and AIDS). Each item is scored from 0 to 6, depending on the stage and severity of the disease (2 points are added for a localized tumor and 6 points for metastases). The maximum possible score is 37, and the score is age-adjusted. To determine the ICC score, we used an online calculator [12]. To optimize data quantification, we established three levels of comorbidity severity: Mild CCI scores between 0 and 3 (0 ≤ CCI scores < 3), Moderate CCI scores between 3 and 5 (3 ≤ CCI scores < 5), and Severe CCI scores of 5 or higher (CCI ≥ 5). These classifications help in understanding the severity of comorbidities more clearly [13,14].

**Figure 3:**
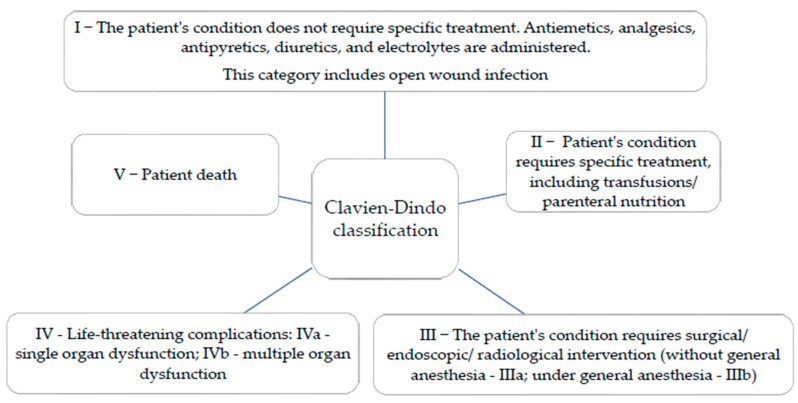
Clavien–Dindo classification.

The complications during hospitalization were quantified using the Clavien–Dindo classification, which stratifies complications according to severity (life-threatening/causing permanent disability), categorized into 5 grades (Figure 3).

### 2.3. Ethical Approval

The study was conducted according to the World Medical Association Declaration of Helsinki guidelines, and it received ethics committee approval (No. 35964/21/11/2024, No. 296/15.01.2025, No. 54150/31.10.2025).

### 2.4. Statistical Analysis

The data were processed using JASP version 0.18.1.0. The mean values of parameters, frequency ranges, and standard deviations were calculated, and statistical significance tests were applied to compare means using the ANOVA method with a significance level set at 0.05. Levene’s test was used to test the homogeneity of the dispersion, and the Shapiro–Wilk test was used to test the normality of the data distribution. If the homogeneity of the dispersion was not observed between the three groups and the variances were found to be significantly different, the Kruskal–Wallis test was used to compare the groups. The chi-square test was used to analyze other distributions between groups. A multiple linear regression model was also implemented to identify predictors.

## 3. Results

### 3.1. Basic Cohort and Group Characteristics

The mean age at the cohort level is 74.74 ± 9.52. Analysis of the data in Table 1 shows that most of the variables compared do not show statistically significant differences between the three groups, except for the environment of origin. Falls trauma from the same level occupies about 90% of the causes leading to PFs. The incidence of PFs at the right hip joint is 63%. The results suggest the prevalence of these fractures in females is over 60% at the cohort level, with no significant differences between groups. The mean interval of occurrence of PFs from THA is 9.33 ± 5.48 years approximately 50% occur within 3–10 years, and 33.33% occur 10 years after primary surgery.

For total hip prostheses, metal alloy endoprostheses (cobalt–chromium, titanium) are used for the femoral heads, and UHMWPE polyethylene for the acetabulum (with ceramic version). The same materials or ceramic components can be used for revisions to reduce wear and improve long-term results. In our study, all endoprostheses used for hip arthroplasty were metal alloy; metal alloy components were also used for revisions.

The data analysis in Table 2 suggests a higher number of THA interventions in group O (*n* = 496) compared to the other study groups. The most frequent cemented THA were present in group B (*n* = 23) vs. 15 in group P and 1 in group O. Regarding the approach, the predominance of the lateral approach is observed in all groups. In group B, the posterior approach was used in about 10% (*n* = 38). Of the total of 54 cases included in the study, 1 case of periprosthetic fracture was associated with a cemented primary prosthesis.

### 3.2. Differences in Average Age/Center

To use statistical analysis assuming equality of variances (ANOVA) further, we applied the test of equality of variances (Levene’s test), which suggests that we can accept the null hypothesis (Table 3), i.e., the variances are equal.

The ANOVA analysis was used to assess whether there are differences between the mean ages of the three groups (75.86 ± 10.93—Group B; 72.78 ± 7.94—Group O; and 74.27 ± 7.36—Group P). The F-value (F = 0.500) and *p*-value (*p* = 0.610) suggest that there is no significant difference between the groups. The *p*-value is higher than the usual significance level, suggesting that there is insufficient evidence to reject the null hypothesis (Table 4).

**Figure 4:**
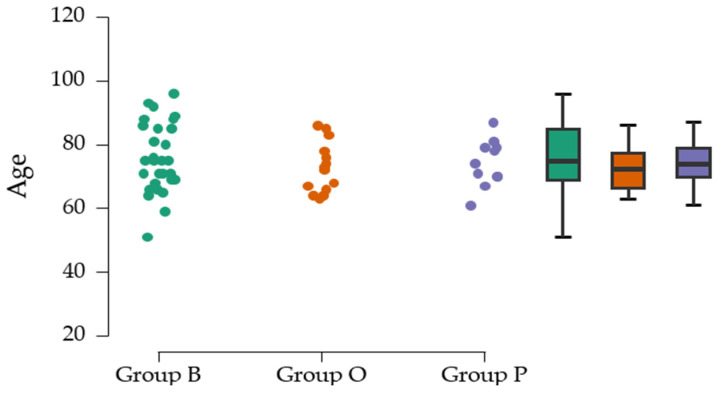
Average age/center.

Figure 4 includes the average age/center.

### 3.3. Differences in Time Interval After THA

**Figure 5:**
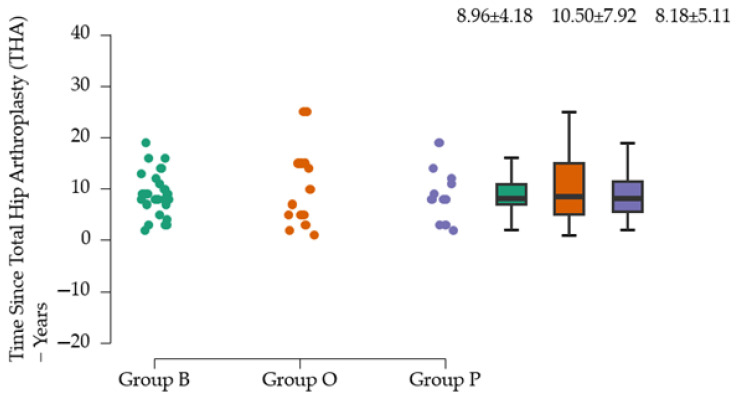
Average time since total hip arthroplasty (THA) in years.

Group O has the highest mean time interval since prosthesis (10.50 ± 7.92), but also the highest variance (75.4%), which may indicate a greater diversity in the responses of subjects in this group (Figure 5). Group B has the lowest relative variance (46.7%), suggesting that distances are more uniform in this group. Group P has a coefficient of 58%.

The *p*-values (*p* = 0.006) and F-values (F = 5.629), obtained when applying Levene’s test of equality of variances, suggest that there is sufficient statistical evidence to reject the null hypothesis that the variances of the groups are equal (Table 5).

For this reason, the Kruskal–Wallis test was used for more than two groups. The results of the Kruskal–Wallis test indicate that the compared groups do not show significant differences in their distributions, as shown by the high *p*-value (Table 6).

### 3.4. The Distribution of PFs According to the Vancouver Classification

The analysis of the data in Table 7 suggests a higher prevalence of PF Vancouver B (over 90%). The prevalence of PFs in patients with cemented hip prostheses is less than 10%/center and about 5% at the cohort level.

### 3.5. Distribution by Type of Intervention/Center

Standard treatment for periprosthetic fractures (PFs) typically involves surgical interventions, including internal fixation or revision arthroplasty. These procedures may involve femoral component replacement and cerclage, depending on the Vancouver classification of the fracture. This classification considers the fracture level, the stability of the prosthesis, and the quality of the surrounding bone. When the femoral component is stable, open reduction and internal fixation are recommended. According to Table 8, the most common intervention is internal fixation, accounting for over 50% of cases both at the cohort level and among the evaluated centers. Conservative treatment was applied to 24.13% of all patients with PFs, with a higher frequency noted in Groups B and P. Group O reported a higher frequency of total arthroplasty revisions (21.42%). The *p*-value of 0.455 indicates that there are no significant differences in therapeutic approaches between the centers.

### 3.6. Patients’ Condition at Discharge

Table 9 suggests that 86.19% of the patients included in the study had a favorable evolution; 5 patients (9.25%) had a stationary evolution, an aspect correlated with the type of intervention (conservative). The incidence of death at the cohort level was 5.55%, but in group B it was 10.34%. The *p*-value = 0.252 suggests that there were no differences in discharge status between centers.

### 3.7. Complications During Hospitalization

The incidence of complications in the cohort was 18.51% (*N* = 10). Complications were predominantly concentrated in group O (28.57%) and group P (18.18%), with group B (13.79%) having fewer complications (Table 10). The *p*-value = 0.093 suggests that there were no differences between centers.

The nature of complications is detailed in Table 11.

Of all patients with PFs, 81.48% had minor complications—grade 1 (Table 12), 9.26% had grade 2 complications (complications requiring medical treatment or other minor interventions), and 3.70% had complications requiring surgery or invasive procedures. Clavien–Dindo grade 5 (patient death) had an incidence of 3.70%. Analysis of the data in the table suggests that the incidence of minor complications predominated in all three study groups. Differences were identified in the higher incidence of grade 2 complications in group O (21.43% vs. 3.45% and 9.09%) and in the higher incidence of grade 5 complications in group B (6.90% vs. 0.00%). The determined *p*-value (*p* = 0.384) suggests the absence of semi-significant differences between groups in the incidence of complications.

### 3.8. Comorbidities Associated with Patients with PFs

Table 13 gives an overview of the diversity and frequency of associated pathologies among the evaluated patients. Cardiac pathology is the most common pathology; hypertension predominates in Group O (42.85%). Alzheimer’s disease was associated in 7 patients (12.96%). Without associated pathology, about 13% of patients were identified. Diabetes mellitus also occurred frequently in 31.50%. The percentage of overweight/obese patients is about 40% at the cohort level.

### 3.9. Charlson Comorbidity Index Assessment/Center

**Figure 6:**
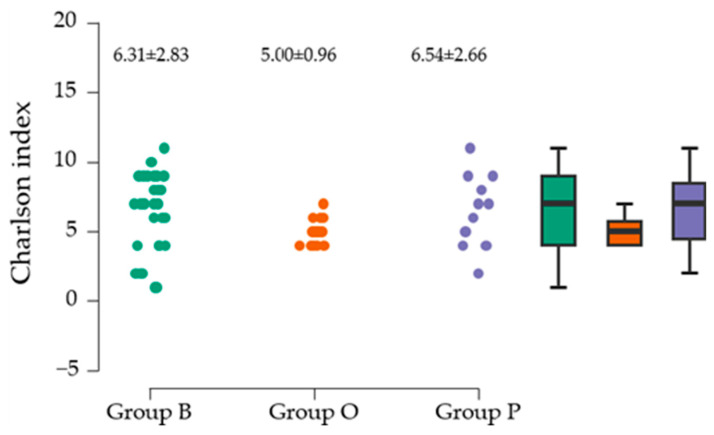
The mean Charlson/center score values.

Data analysis indicated that the CCI, at the cohort level, has a mean of 6.019 ± 2.48, with moderate variability, and the distribution of values is shown to be normal (*p* = 0.965). The values range from 1 to 11, suggesting a diversity of comorbidity levels in the sample. The mean Charlson/center score values are contained in Figure 6, which suggests that the highest mean was associated with group P, with moderate variability of scores, compared to the high variability of scores found in group B. In the study, all CCI means (overall and by center) are above 5. A value ≥ 5 suggests that patients have significant comorbidities that greatly increase the risk of mortality.

Because the *p*-value (0.002) obtained with Levene’s test is less than the common alpha level of 0.05, we reject the null hypothesis regarding the distribution of variances (Table 14).

Additionally, the Kruskal–Wallis test was conducted, indicating insufficient evidence to support significant differences among the three groups in the Charlson Comorbidity Index (Table 15).

### 3.10. The Association of the Charlson Comorbidity Index with the Clavien–Dindo Index

The data presented in Table 16 indicate a very weak positive correlation between the Dindo Index and the CCI (r = 0.046), which is not statistically significant (*p* = 0.628). The effect size, measured by Fisher’s z, is also reported as 0.046. Given that the value of r is very small, the effect size further suggests that the relationship between the two indices is negligible.

Furthermore, the association between Clavien–Dindo Complications Index and age was tested. Data analysis in Table 17 shows that there is a weak correlation and it cannot be considered statistically relevant.

## 4. Discussion

The aim of the study was to describe the incidence of PFs, immediate postoperative complications, and comorbidities in patients with PFs from three emergency hospitals in Romania.

The prevalence of PFs is increasing, as predicted by Della Rocca et al., based on increasing life expectancy and the increasing number of total hip arthroplasties [15]. The study published by Minutillo GT (2024) emphasizes the increase in the incidence of periprosthetic hip fractures from 2016 to 2021 by 38% and estimates an increase in periprosthetic fractures, in general, of 212% in 2032 (compared to 2016) (5). In our study, the frequency of these fractures is different according to the evaluated center. Thus, in Group O, the frequency is 2.82% vs. 7.75% (group B) and 5.39% (group P); the explanation could be the increased addressability in the university center Bucharest. At the cohort level, the prevalence of FPs is 5.02%—higher than in other studies [3].

The mean age at the cohort level is 74.74 ± 9.52 years and is comparable with other results published in the literature [4], with no significant differences in mean age between the three groups evaluated. The most common mechanism of periprosthetic fractures (PFs) in our study was falls, with a frequency of 88.89%. Literature data support this main mechanism but indicate a lower frequency of 76% [3]. In groups P and O, injuries caused by falls from the same level are the only cause identified. According to our analysis, motor vehicle accidents account for about 10% of all identified causes of PFs. Our study also suggests that about 50% of PFs occur within 3–10 years after THA. The incidence of PFs in women is higher in our study (62.94%) and in similar studies, where an incidence of 66% was reported [16]. Group O has the highest total number of THA interventions, which can be explained by the high number of patients from neighboring counties. Group P has the lowest total number of interventions, which is due to the fact that it is located close to other hospitals in the capital city. The surgical approach for implanting the primary prosthesis was predominantly lateral; in group B, the posterior approach was chosen for intervention in 10%.

Data analysis suggests a low percentage of cemented prosthesis use (less than 5%), which can be explained by the fact that studies have shown that uncemented revision arthroplasty is more effective, as it reduces the risks related to complications caused by cement extrusion [17]. Cement fixation was an important risk factor for periprosthetic fractures, as individuals with cemented implants had a reduced likelihood of experiencing such fractures [5,18]. However, the multicenter PIPPAS study published in 2024 (*n* = 1387) suggests that THA-associated PFs occurred equally in cemented and uncemented strains [1], in contrast to our study, in which fractures occurring on cemented prostheses were 5.55%. Also in this study, a higher rate of THA-associated PFs (6.2%) compared to PFs with other localizations is reported.

Most PFs belonged to the Vancouver B classification, similar to the results obtained by other authors [16]. In terms of therapeutic management, changes in favor of open reduction and internal fixation versus revision arthroplasty have been reported [19,20]. Given this Vancouver classification, the predominant surgical technique approached in this study was internal fixation with cerclage (more than 50%). Data analysis suggests the absence of significant differences between the centers evaluated in terms of therapeutic approach technique, incidence of postoperative complications, and discharge status.

Most complications across all three groups are classified as low severity (index 1). However, Group O shows a higher incidence of moderate severity complications (indexes 2 and 3) compared to Group B. Group P exhibits a similar profile to Group B regarding minor complications, but with a slightly higher frequency of moderate complications. Indexes 4 and 5 are very rare, indicating effective management of complications within these groups.

Another objective of the research was to establish the relationship between preoperative comorbidities, analyzed using the CCI, a tool recognized for its validity in assessing comorbidities, and immediate postoperative complications. The most common associated comorbidities were diabetes mellitus and cardiac disease. Cardiovascular diseases associated with PFs in our study are present in more than 90% of patients. The mean value of the CCI did not differ significantly between the three groups evaluated. All three deaths in the study were associated with a CCI above 5, which correlates with the results of the multicentric PIPPAS study [1]. The study published by B.Y. Park (2019) [21] aimed to correlate comorbidities with postoperative complications in 110 THA patients. The association of superficial infections in patients with diabetes was found [21]. Our study reports a death incidence of 3.70%, which is higher than the 2.5% reported by JN Lamb et al. in 2022 (*n* = 4841) [6].

The three deaths (two women) all occurred in group B, and the age of the patients was over 85 years. This trend can be attributed to several factors, including the patients’ age (all over 85 years), the larger patient population, and the complexity of cases referred to the Bucharest Emergency Clinical Hospital, one of the largest hospitals in Romania. The incidence of death in group B (10.34%) is twice the value published by Nasser A.A. et al. (2023) [22]. Associated comorbidities were heart failure, stroke sequelae, a pacemaker, and diabetes mellitus. A higher CCI suggests greater frailty, which may partly contribute to an increased risk of periprosthetic fracture [23].

The study published by J. A. Singh and D.G. Lewallen (*n* = 20,346) suggests an association between peptic ulcer disease and heart disease in patients with primary THA and revision THA with postoperative periprosthetic fracture [24]. In our study, there is insufficient evidence to suggest that the CCI and the Clavien–Dindo Index are significantly associated. Longer-term studies are needed to follow these associations. The retrospective study published by S Märdian et al. (2017, *n* = 151 patients with PFs) [23] tracked the survival of patients with PFs relative to the general population. The results support the association of mortality in patients who had previous cardiovascular disease comorbidities (ischemic heart disease, cardiac arrhythmias, and heart failure) [23].

### The Strengths and Limitations of the Study

One of the main strengths of the study is that it is, to our knowledge, the only multicenter study in Romania to date with a significant number of cases, which makes it of high value in the research context. However, an important limitation is the varying sample sizes, which may influence the interpretation of the data, in particular in terms of standard error and coefficient of variation.

## 5. Conclusions

The data analysis indicates that the main cause of periprosthetic fractures associated with total hip arthroplasty is falls, emphasizing the importance of fall prevention among elderly patients. In addition, the majority of fractures occur 3 to 10 years after prosthetic surgery, and the incidence is higher in women. There was a higher incidence of deaths among elderly patients with comorbidities such as diabetes and heart disease, emphasizing the need for increased attention in the management of cases with comorbidities. However, no significant differences in therapeutic approaches or postoperative complications were observed between the evaluated centers. These findings indicate an urgent need to update treatment and prevention protocols, especially given the anticipated increase in peri-prosthetic fractures in the future. In this context, it is essential to develop effective strategies for the management of patients with total hip arthroplasty aimed at minimizing risks and improving long-term outcomes.

## Figures and Tables

**Table 1:** Basic cohort and group characteristics.

Parameter		Cohort	Group B	Group O	Group P	*p*-Value
Average age, M, SD		74.74 ± 9.52	75.86 ± 10.94	72.78 ± 7.94	74.27	0.610
Environment of origin, *N* (%)	urban	19 (64.81)	8 (27.59)	9 (64.29)	2 (18.182)	0.026
Sex, *N* (%)	women	34 (62.94)	18 (62.07)	8 (57.15)	8 (72.72)	0.718
Hip joint involvement, *N* (%)	right	34 (62.96)	20 (68.96)	8 (57.14)	6 (54.55)	0.611
	left	20 (37.03)	9 (31.03)	6 (42.85)	5 (45.45)	
Etiology, *N* (%)	car accident	4 ((7.40)	4 (13.78)	0 (0.00)	0 (0.00)	0.668
	skiing accident	1 (1.85)	1 (3.44)	0 (0.00)	0 (0.00)	
	fall	48 (88.89)	23 (79.31)	14 (100.0)	11 (100.0)	
	crushing	1 (1.85)	1 (3.44)	0 (0.00)	0 (0.00)	
Time interval from THA, *N* (%)	over 10 years	18 (33.33)	8 (27.58)	6 (42.86)	4 (36.36)	0.559
	between 3 and 10 years	26 (48.15)	17 (58.62)	5 (35.71)	4 (36.36)	
	under 3 years	10 (18.20)	4 (13.80)	3 (21.43)	3 (21.43)	
Discharge status, *N* (%)	improved	46 (85.18)	23 (79.31)	14 (100.0)	9 (81.81)	0.252
	exitus	3 (5.55)	3 (10.34)	0 (0.00)	0 (0.00)	
	stationary	5 (9.26)	3 (10.34)	0 (0.00)	2 (18.18)	

**Table 2:** Number of hip arthroplasty/year/center, number of hip arthroplasty/year/center, and surgical approach.

Parameter		Group B	Group O	Group P	Total
THA total/year		374	496	204	1074
THA cemented		23	1	15	39
Surgical approach	lateral	336	496	204	1036
	posterior	38	0	0	38

**Table 3:** Test for equality of variances (Levene’s).

F	df1	df2	*p*
1.658	2.000	51.000	0.201

**Table 4:** The ANOVA analysis of the differences between the means of the ages of the three study groups.

Cases	Sum of Squares	df	Mean Square	F	*p*
GROUP	92.383	2	46.192	0.500	0.610
Residuals	4711.987	51	92.392		

**Table 5:** Test for equality of variances (Levene’s).

F	df1	df2	*p*
5.629	2.000	51.000	0.006

**Table 6:** Kruskal–Wallis test to determine differences between mean within-interval times after THA implantation.

Factor	Statistic	df	*p*
Group	0.076	2	0.963

**Table 7:** The distribution of PFs according to the Vancouver classification.

Parameter	Group B	Group O	Group P	Cohort
PFs occurring on cemented THAs	1 (3.44)	1 (7.14)	1 (9.09)	3 (5.55)
PFs Vancouver A, *N* (%)	1 (3.45)	0 (0.00)	2 (18.18)	3 (8.47)
PFs Vancouver B, *N* (%)	28 (96.55)	14 (100.00)	9 (81.82)	51 (91.53)

**Table 8:** Therapeutic management by centers.

Intervention	Group B	Group O	Group P	Cohort
Internal fixation, *N* (%)	17 (58.62)	8 (57.14)	6 (54.54)	28 (51.85)
Conservative, *N* (%)	7 (24.13)	2 (14.28)	4 (36.36)	12 (22.22)
Closed fracture reduction and fixation with external fixator, *N* (%)	1 (3.44)	0 (0.00)	0 (0.00)	1 (1.85)
Partial arthroplasty revision, *N* (%)	3 (10.34)	1 (7.14)	1 (9.09)	5 (9.25)
Total arthroplasty revision, *N* (%)	1 (3.44)	3 (21.42)	0 (0.00)	4 (7.40)

**Table 9:** Distribution of discharge status/center.

Discharge Status	Group B	Group O	Group P	Cohort
Improved, *N* (%)	23 (79.31)	14 (100.0)	9 (81.81)	46 (86.19)
Exitus, *N* (%)	3 (10.34)	0 (0.00)	0 (0.00)	3 (5.55)
Stationary, *N* (%)	3 (10.34)	0 (0.00)	2 (18.18)	5 (9.25)

**Table 10:** Number of complications occurring during hospitalization/center.

Group	Complications, *N* (%)	Without Complications, *N* (%)
Group B	4 (13.79)	25 (86.21)
Group O	4 (28.57)	10 (71.2)
Group P	2 (18.18)	9 (81.81)
Cohort	10 (18.51%)	44 (81.48)

**Table 11:** Complications during hospitalization.

Group	Complications	Frequency	Percent
Group B	Exitus	3	10.34
	Bed Sores	1	3.448
	Phlebitis	0	0.000
	Phlebitis, Urinary Infection	0	0.000
	Bleeding	0	0.000
	Pulmonary Thromboembolism	1	3.448
Group O	Exitus	0	0.000
	Bed sores	0	0.000
	Phlebitis	3	21.429
	Phlebitis, Urinary Infection	1	7.143
	Bleeding	0	0.000
	Pulmonary Thromboembolism	0	0.000
Group P	Exitus	0	0.000
	Bed Sores	1	9.091
	Phlebitis	0	0.000
	Phlebitis, Urinary Infection	0	0.000
	Bleeding	1	9.091
	Pulmonary Thromboembolism	0	0.000

**Table 12:** The frequency of complications evaluated with the Clavien–Dindo Index.

Clavien–Dindo Index	Group B, *N* (%)	Group O, *N* (%)	Group P, *N* (%)	Cohort, *N* (%)
1	25 (86.20)	10 (71.43)	9 (81.81)	44 (81.48)
2	1 (3.45)	3 (21.43)	1 (9.09)	5 (9.26)
3	0 (0.00)	1 (7.14)	1 (9.09)	2 (3.70)
4	1 (3.45)	0 (0.00)	0 (0.00)	1 (1.85)
5	2 (6.90)	0 (0.00)	0 (0.00)	2 (3.70)

**Table 13:** Frequency of associated pathology/center/cohort.

Associated Pathology	Group B *N* (%)	Group O *N* (%)	Group P *N* (%)	Cohort *N* (%)
Alzheimer’s Disease	2 (6.90)	0 (0.00)	0 (0.00)	2 (3.70)
Coronary Heart Disease, Hypertension	1 (3.44)	0 (0.00)	0 (0.00)	1 (1.85)
Sinus Bradycardia	0 (0.00)	1 (7.14)	0 (0.00)	1 (1.85)
Diabetes Mellitus	4 (13.80)	0 (0.00)	0 (0.00)	4 (7.40)
Diabetes Mellitus, Hypertension	2 (6.89)	1 (7.15)	0 (0.00)	3 (5.59)
Diabetes Mellitus, Hypertension, Hydrostatic Varices	1 (3.45)	0 (0.00)	0 (0.00)	1 (1.85)
Diabetes Mellitus, Sequelae Of Stroke	0 (0.00)	0 (0.00)	1 (9.09)	1 (1.85)
Diabetes Mellitus, Hypertension, Alzheimer’s Disease	0 (0.00)	0 (0.00)	1 (9.01)	1 (1.85)
Diabetes Mellitus, Heart Failure	2 (6.90)	0 (0.00)	2 (18.18)	4 (7.40)
Diabetes Mellitus, Heart Failure, Hypertension	1 (3.45)	0 (0.00)	1 (9.01)	2 (3.70)
Hypertension	1 (3.45)	6 (42.85)	3 (27.27)	10 (18.51)
Hypertension, Alzheimer’s Disease	1 (3.45)	0 (0.00)	0 (0.00)	1 (1.85)
Hypertension, Alzheimer’s Disease, Ischemic Heart Disease	0 (0.00)	1 (7.15)	0 (0.00)	1 (1.85)
Hypertension, Alzheimer’s Disease, Diabetes Mellitus	1 (3.45)	0 (0.00)	0 (0.00)	1 (1.85)
Hypertension, Diabetic, Hypothyroidism	0 (0.00)	1 (7.15)	0 (0.00)	1 (1.85)
Hypertension, Hypothyroidism	0 (0.00)	1 (7.15)	0 (0.00)	1 (1.85)
Hypertension, Chronic Kidney Disease, Heart Failure, Aortic Aneurysm	1 (3.45)	0 (0.00)	1 (9.01)	2 (3.70)
Hypertension, Alzheimer’s Disease	0 (0.00)	0 (0.00)	1 (9.01)	1 (1.85)
Hypertension, Valvular Insufficiency, TB Sequelae, Diabetes Mellitus	1 (3.45)	0 (0.00)	0 (0.00)	1 (1.85)
Hypertension, Psoriasis	1 (3.45)	0 (0.00)	1 (9.01)	2 (3.70)
Myocardial Infarction, Ischemic Heart Disease, Coronary Stent, Hypertension	0 (0.00)	1 (7.15)	0 (0.00)	1 (1.85)
Valvular Insufficiency	1 (3.45)	0 (0.00)	0 (0.00)	1 (1.85)
Sequelae Of Stroke, Alzheimer’s Disease, Hypertension	1 (3.45)	0 (0.00)	0 (0.00)	1 (1.85)
Sequelae Of Stroke, Heart Failure, Hypertension	2 (6.90)	0 (0.00)	0 (0.00)	2 (3.70)
Stroke Sequelae, Heart Failure, Cardiac Implantation, Cardiac Implant	1 (3.45)	0 (0.00)	0 (0.00)	1 (1.85)
Overweight/obese	13 (44.82)	6 (42.85)	3 (27.27)	22 (40.70)
N.A.	5 (17.25)	2 (14.28)	0 (0.00)	7 (12.96)

N.A.—without associated complications.

**Table 14:** Levene’s test of equality of variances.

F	df1	df2	*p*
6.993	2.000	51.000	0.002

**Table 15:** Kruskal–Wallis test.

Factor	Statistic	df	*p*
Group	4.521	2	0.104

**Table 16:** The correlation of the CCI with the Clavien–Dindo Complications Index.

Variable	Parameter	Dindo Index
CCI	Pearson’s r	0.046
	*p*-value	0.628
	Effect size (Fisher’s z)	0.046
	SE effect size	0.140

**Table 17:** Correlation of age with the Clavien–Dindo Complications Index.

Variable	Parameter	Dindo Index
Age	Pearson’s r	0.125
	*p*-value	0.367
	Effect size (Fisher’s z)	0.126
	SE effect size	0.140

## Data Availability

The data presented in this study are available on request from the first author.
